# Proteasome Inhibitor MG132 is Toxic and Inhibits the Proliferation of Rat Neural Stem Cells but Increases BDNF Expression to Protect Neurons

**DOI:** 10.3390/biom10111507

**Published:** 2020-11-02

**Authors:** Young Min Kim, Hyun-Jung Kim

**Affiliations:** College of Pharmacy, Chung-Ang University, Seoul 06974, Korea; cholove_00@naver.com

**Keywords:** protein degradation, MG132, neural stem cells, neurogenesis

## Abstract

Regulation of protein expression is essential for maintaining normal cell function. Proteasomes play important roles in protein degradation and dysregulation of proteasomes is implicated in neurodegenerative disorders. In this study, using a proteasome inhibitor MG132, we showed that proteasome inhibition reduces neural stem cell (NSC) proliferation and is toxic to NSCs. Interestingly, MG132 treatment increased the percentage of neurons in both proliferation and differentiation culture conditions of NSCs. Proteasome inhibition reduced B-cell lymphoma 2 (Bcl-2)/Bcl-2 associated X protein ratio. In addition, MG132 treatment induced cAMP response element-binding protein phosphorylation and increased the expression of brain-derived neurotrophic factor transcripts and proteins. These data suggest that proteasome function is important for NSC survival and differentiation. Moreover, although MG132 is toxic to NSCs, it may increase neurogenesis. Therefore, by modifying MG132 chemical structure and developing none toxic proteasome inhibitors, neurogenic chemicals can be developed to control NSC cell fate.

## 1. Introduction

Neural stem cells (NSCs) possess the capacity to self-renew and can differentiate into neurons, astrocytes, and oligodendrocytes [1,2,3]. NSCs are found in vivo, such as in the developing brain and in specific regions of the adult brain [1,4,5]. NSCs are good tools to study brain regeneration and can be therapeutically used to replace degenerated neurons and glia in various neurodegenerative diseases [1,5,6,7]. Thus, it is critical to understand the regulation mechanism of NSC fate.

Different types of cells have cell-type-specific protein profile expression; moreover, gene transcription, translation, and protein degradation regulate the distinct proteome expression [2,8,9,10]. Protein breakdown occurs in the lysosomes by acid hydrolases and in the proteasomes by an ATP-dependent mechanism [11]. The eukaryotic 26S proteasome is a 2.5 MDa multi-catalytic proteinase complex composed of a 19S regulatory unit and a 20S core unit [11,12]. The 19S regulatory particle caps one or both sides of the 20S core unit and controls the entrance of degradable proteins to the 20S particle, which has proteolytic activities [13]. Proteins prone to degradation are modified by ubiquitin and recognized by proteasomes [14].

Dysregulation of proteasomes is implicated in various diseases, including cancers and neurodegenerative disorders [15]. It was reported that cancer cells use proteasome activity for their survival and proliferation; therefore, proteasome inhibitors are developed to cure cancers [16,17,18,19,20,21,22]. For example, the proteasome inhibitor bortezomib is the first choice of drug to treat multiple myeloma [16,17,22,23,24,25]. Although protein aggregates may be too large to be broken up in the proteasomes, and much attention has been paid to autophagy-lysosomal protein degradation, it would be interesting to explore the roles of proteasomes in the neural system.

MG132 is an effective peptide aldehyde proteasome inhibitor (carbobenzoxyl-l-leucyl-l-leucyl-leucine) that has been extensively studied [26,27,28,29,30,31]. Non-functional, excessive, misfolded, and waste proteins should be removed from cells, but aging or other pathological conditions result in decreased proteasome activity and lead to neurodegenerative diseases [15]. In the current study, we used MG132 to inhibit proteasome activity and investigated the role of proteasomes in NSC proliferation, differentiation, and survival.

## 2. Materials and Methods

### 2.1. Reagents

The proteasome inhibitor MG132 was purchased from Calbiochem (474790; San Diego, CA, USA). MG132 was dissolved in dimethyl sulfoxide (DMSO) (472301; Sigma-Aldrich, Saint Louis, MO, USA) at 10 mM as a stock solution. DMSO was used as a control vehicle, and MG132 stock solution was kept at −20 °C. 

### 2.2. NSC Culture

NSCs were cultured as previously described [32]. Animal experiments were approved by Chung-Ang University animal care and use committee and performed in accordance with Chung-Ang University and NIH standards of animal care (permission number: 13-0049, 2014-00032). Mycoplasma tests were not performed. Briefly, NSCs were isolated from the cortex of E14 Sprague−Dawley rat embryos (Orient Bio Inc.,Seongnam-si, Gyeonggi-do, Korea), seeded onto tissue culture flasks (200,000 cells/mL), and expanded as neurospheres for 7 days in Dulbecco’s modified Eagle medium/F12 supplemented with 1% (*v*/*v*) antibiotic-antimycotic, 2% (*v*/*v*) B27 (15240-062; 17504-044; all from Thermo Fisher Scientific, Waltham, MA, USA), and 20 ng/mL each of epidermal growth factor (EGF) and fibroblast growth factor 2 (FGF2) (GF144; GF003AF; all from Merck Millipore, Burlington, MA, USA). The medium was replaced every 2 days, and the culture was maintained at 37 °C in 5% CO_2_. After 6 days, neurospheres were dissociated into a single-cell suspension with accutase (SCR005; Chemicon, Temecula, CA, USA) for 10 min at 37 °C and plated onto 0.01% poly-d-lysine (P0899; Sigma-Aldrich, MO, USA) and 10 μg/mL laminin (23017-015; Invitrogen, Carlsbad, CA, USA)-coated plates, and expanded for 1 day. After 1 day of proliferation, differentiation was induced in the absence of growth factors. For the experiments, NSCs were treated with 0.1% DMSO, 10 ng/mL ciliary neurotrophic factor (CNTF), or MG132.

### 2.3. Cell Viability Assay

Cell viability was assessed using the 3-(4,5-dimethylthiazol-2-yl)-2,5-diphenyltetrazolium bromide (MTT) (M5655; Sigma-Aldrich, Saint Louis, MO, USA) assay [33,34]. After neurosphere expansion for 1 week, NSCs were plated onto 48-well plates (Corning, Corning, NY, USA) for 24 h followed by DMSO or MG132 treatment for 48 h. MTT solution (1 mg/mL) was added to each well, and the cells were incubated for 2 h at 37 °C. The formazan crystals that formed in NSCs were solubilized with 20% sodium dodecyl sulfate (SDS) (0227; Amresco, Solon, OH, USA) in 50% aqueous N,*N*-dimethylformamide (D4551; Sigma-Aldrich, Saint Louis, MO, USA). Lysates were transferred to 96-well plates (Corning, Corning, NY, USA), and the absorbance was measured at 550 nm using a Synergy H1 Hybrid Multi-Mode Microplate Reader (Biotek, Winooski, VT, USA).

### 2.4. Neurosphere Growth Rate

Neurosphere growth was measured as previously described [33,34,35,36]. Briefly, individual spheres (approximately 80–110 μm in diameter) were transferred to a single well in 96-well plates (*n* = 3) containing 200 μL culture medium that promotes proliferation. Neurospheres were treated with DMSO or MG132, and the diameter of the neurospheres was measured daily using a lens-mounted microscope (Leica, Wetzlar, Germany). The volume of each neurosphere was calculated by the equation *V* = 4/3πr^3^, *r* = 1/2 diameter, as described previously [37]. Neurosphere volume on each day (day 0–4) was divided by the volume on day 0 and then multiplied by 100 to obtain the percentage increase in sphere volume.

### 2.5. Western Blot Analysis

NSCs were washed with phosphate-buffered saline (PBS) and lysed in a buffer [5 mM EDTA, 50 mM HEPES, 50 mM NaCl, 1% NP-40, 1% Triton X-100 (all from Amresco, Solon, OH, USA), Halt Phosphatase Inhibitor Cocktail (78420; Thermo-Fisher Scientific, Rockford, IL, USA), 1 mM phenylmethylsulfonyl fluoride, 0.01 mg/mL aprotinin, and 0.01 mg/mL leupeptin (P7626; A1153; L9783; all from Sigma-Aldrich, Saint Louis, MO, USA)]. Cell lysates were centrifuged for 20 min at 25,200× *g* to remove debris and boiled for 5 min in SDS sample buffer 60 mM Tris-HCl of pH 6.8, 25% glycerol, 2% SDS, 0.1% bromophenol blue (all from Amresco, Solon, OH, USA), 14.4 mM β-mercaptoethanol (M6250; Bio-Rad, Hercules, CA, USA), and distilled water. The proteins were separated electrophoretically on an SDS-polyacrylamide gel and transferred to polyvinylidene fluoride membranes (Merck Millipore, Burlington, MA, USA) for 1 h [GangNam-STAIN™ Prestained Protein Ladder (24052; iNtRON Biotechnology, Gyeonggi-do, Korea), Precision Plus Protein™ Dual Color Standards (#161-0374; Bio-Rad, Hercules, CA, USA)]. The membranes were blocked in 5% skim milk or bovine serum albumin (82-100-6; Merck Millipore, Burlington, MA, USA) in 20 mM Tris-buffered saline containing 0.03−0.1% Tween 20 (M147; Amresco, Solon, OH, USA) for 1 h and incubated overnight at 4 °C with primary antibodies: anti-glial fibrillary acidic protein (GFAP) (1:500, G9269), TuJ1 (1:1000, T5076; all from Sigma-Aldrich, Saint Louis, MO, USA), phospho-cAMP response element-binding protein (pCREB) (1:1000, #06-519; Merck Millipore, Burlington, MA, USA), brain-derived neurotrophic factor (BDNF) (1:1000, ab108319; Abcam, Cambridge, MA, USA), B-cell lymphoma 2 (Bcl-2) (1:100, sc-7382; Santa Cruz, Dallas, TX, USA), Bcl-2 associated X protein (Bax) (1:1000, #2772; Cell Signaling, Danvers, MA, USA), and glyceraldehyde 3-phosphate dehydrogenase (GAPDH) (1:1000, sc-32233; Santa Cruz, Dallas, TX, USA), followed by horseradish peroxidase-conjugated secondary antibodies: anti-rabbit IgG (1:5000, ADI-SAB-300-J) or anti-mouse IgG (1:5000, ADI-SAB-100-J; all from Santa Cruz, Dallas, TX, USA). Protein bands were detected on a CP-BU New X-ray film (Agfa, Mortsel, Belgium) using Western Blotting Luminol Reagent (Santa Cruz, Dallas, TX, USA).

### 2.6. Real-Time Reverse Transcription Polymerase Chain Reaction (qRT-PCR)

The total RNA was isolated from cells with TRIzol reagent (15596026; Invitrogen, Carlsbad, CA, USA). First-strand complementary DNA (cDNA) was synthesized from 1 μg of total RNA using a QuantiTect Reverse Transcription Kit (Qiagen, Venlo, Limburg, Netherlands). qRT-PCR was performed with iQ™ SYBR Green supermix (#170-8882AP; Bio-Rad, Hercules, CA, USA). The following primer sets were used to amplify cDNA: *Bdnf*, gcccaacgaagaaaaccataag (forward) and gtttgcggcatccaggtaatt (reverse); *Bcl-2*, tgacttctctcgtcgctacc (forward) and gaactcaaagaaggccacaa (reverse); *Bax*, tggttgcccttttctactttg (forward) and gaagtaggaaaggaggccatc (reverse); and *Gapdh*, agttcaacggcacagtcaag (forward) and gtggtgaagacgccagtaga (reverse). The qRT-PCR conditions were as follows: initial activation at 95 °C for 3 min followed by 40 cycles of denaturation at 95 °C for 10 s, annealing at 58 °C for 15 s, and extension at 72 °C for 20 s. The housekeeping gene *Gapdh* was used as an internal control.

### 2.7. Immunocytochemistry (ICC) and Cell Counting

ICC analysis was performed as previously described [38,39,40]. Cell cultures were fixed using 4% paraformaldehyde (SC-281692; USB Products, Cleveland, OH, USA) for 30 min and washed with PBS. Fixed cells were blocked with 5% normal goat serum (S26; Merck Millipore, Burlington, CA, USA) and 0.2% Triton X-100 (M143; Amresco, Solon, OH, USA) in PBS for 30 min and incubated with primary antibodies: anti-GFAP (rabbit IgG, 1:1000, Z0334; Agilent, Santa Clara, CA, USA), TuJ1 (mouse IgG2b, 1:1000, T5076; Sigma-Aldrich, Saint Louis, MO, USA), and Ki67 (rabbitIgG, 1:400, RM-9106-S0; Thermo Fisher Scientific, CA, USA) for 1 h 30 min. After rinsing with PBS, the cells were incubated with secondary antibodies conjugated to Cy3 (goat anti-rabbit IgG, 1:1000, 111-165-144; Jackson ImmunoResearch, West Grove, PA, USA) or Alexa Fluor 488 (goat anti-mouse IgG, 1:1000, A11001; Thermo Fisher Scientific, CA, USA) for 30 min, followed by the addition of 4′,6-diamidino-2-phenylindole (DAPI) (1:10,000, D9542, in PBS; Sigma-Aldrich, Saint Louis, MO, USA) to stain the nuclei for 5 min. The images were obtained using an inverse fluorescence microscope (Leica, Hesse, Germany). To avoid measurement bias, photos were taken in three randomly selected microscopic fields, and TuJ1-, GFAP-, Ki67-, or DAPI-positive cells were counted. The number of TuJ1-, GFAP-, or Ki67-positive cells was normalized to the total number of DAPI-positive cells. The value of the MG132-treated group was divided by that of the control group to obtain the fold change.

### 2.8. Statistical Analysis

Values are expressed as means ± standard error of mean or mean ± standard deviation, and statistical significance was determined using Student’s *t*-test (* *p* < 0.05, and ** *p* < 0.01).

## 3. Results

### 3.1. MG132 Increases Neuron Percentage in Rat NSCs in the Presence of Growth Factors

**Figure 1:**
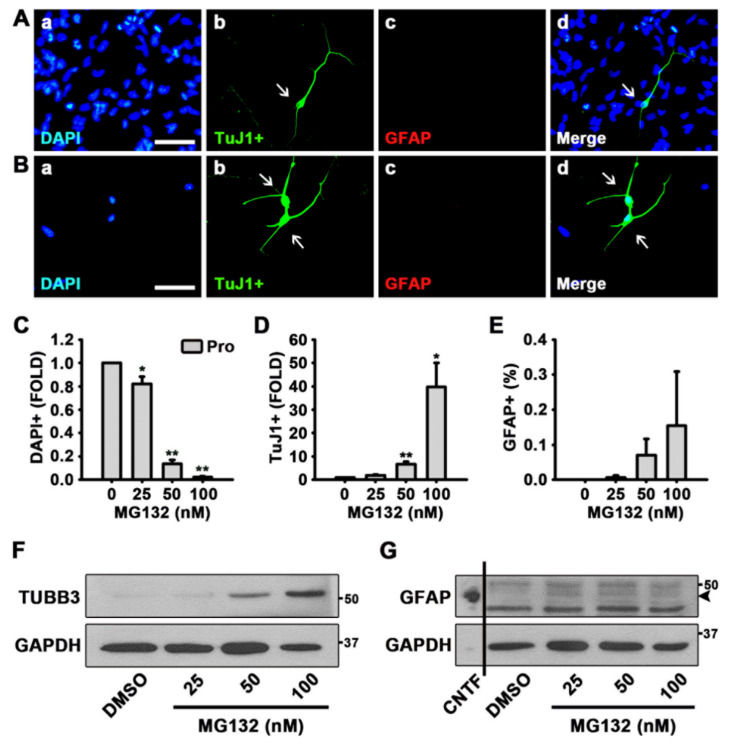
MG132 increases the percentage of neurons in the presence of growth factors in neural stem cells (NSCs). (**A**,**B**) Representative immunofluorescence images of NSCs proliferated for 48 h [4′,6-diamidino-2-phenylindole (DAPI)-positive nuclei (**a**, blue), TuJ1-positive neurons (**b**, green), glial fibrillary acidic protein (GFAP)-positive astrocytes (**c**, red), and merged image (**d**)]. Scale bar = 50 µm. NSCs were treated with (**A**) dimethyl sulfoxide (DMSO) or (**B**) 100 nM MG132 in the presence of growth factors. (**C**–**E**) Quantification of (**C**) nuclei, (**D**) neurons, and (**E**) astrocytes. Data are expressed as mean ± standard error of mean (SEM) (*n* = 3). (**F**,**G**) Representative images of the protein bands of (**F**) βIII Tubulin (TUBB3) and (**G**) GFAP. Two days after treatment, total cell lysates from proliferated NSCs were subjected to western blotting analysis with TuJ1 and GFAP antibodies. Glyceraldehyde 3-phosphate dehydrogenase (GAPDH) was used as a loading control, and ciliary neurotrophic factor (CNTF) was used as a positive control for GFAP. * *p* < 0.05, and ** *p* < 0.01 (Student’s *t*-test).

To explore the role of protein degradation in the control of NSC fate, we treated NSCs with a proteasome inhibitor, MG132, for 48 h in the presence of mitogens, EGF, and FGF2. NSCs are known to proliferate in the presence of growth factors and differentiate into neurons or glia in the absence of growth factors [39,40,41,42]. The ICC data revealed that 100 nM MG132 significantly reduced cell proliferation and induced the percentage of neurons in NSCs, even in the presence of EGF and FGF2 (Figure 1A,D). Neurons of DMSO- or MG132 (100 nM)-treated NSCs were detected by TuJ1 antibody that recognizes βIII Tubulin (TUBB3) (Figure 1A,B). As shown in Figure 1C,D, MG132 significantly reduced the number of cells and increased the percentage of neurons in a concentration-dependent manner. Astrocytes were determined by the antibody that detects GFAP (Figure 1A,B); however, quantitation results showed that MG132 does not significantly enhance astrocytogenesis (Figure 1E). Western blotting data confirmed the induction of TUBB3 expression without induction effect on GFAP expression by MG132 in NSCs in the presence of mitogens (Figure 1F,G and Figure S1).

**Figure 2:**
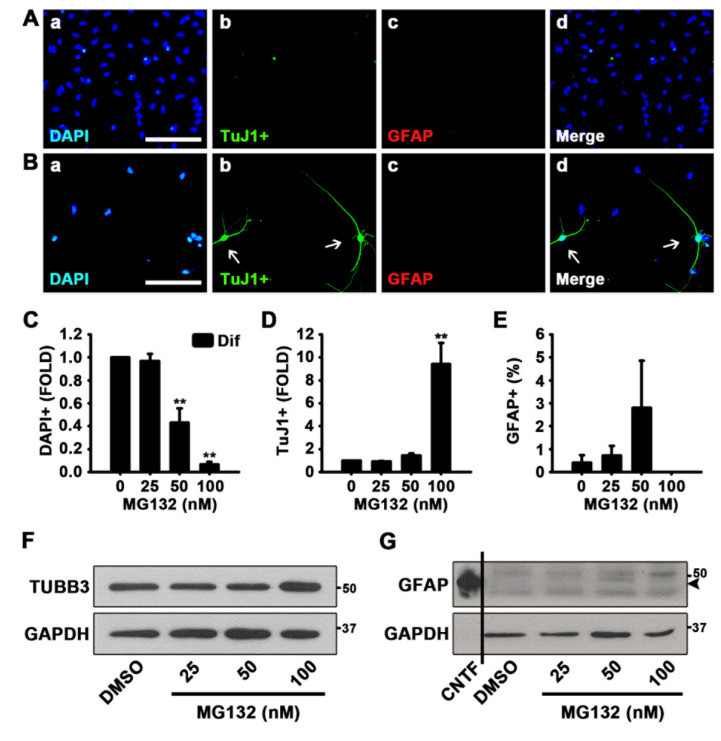
MG132 increases the percentage of neurons in the absence of growth factors. (**A**,**B**) Representative immunofluorescence images of NSCs differentiated for 48 h DAPI-positive nuclei (**a**, blue), TuJ1-positive neurons (**b**, green), GFAP-positive astrocytes (**c**, red), and merged image (**d**). Scale bar = 100 µm. NSCs were treated with (**A**) DMSO and (**B**) 100 nM MG132 in the absence of epidermal growth factor (EGF) and fibroblast growth factor 2 (FGF2). (**C**–**E**) Quantification of (**C**) nuclei, (**D**) neurons, and (**E**) astrocytes. Data are expressed as mean ± SEM (*n* = 3). (**F**,**G**) Representative images of the protein bands of (**F**) TUBB3 and (**G**) GFAP. Two days after treatment, total cell lysates from differentiated NSCs were subjected to western blotting analysis with TuJ1 and GFAP antibodies. GAPDH was used as a loading control, and CNTF was used as a positive control for GFAP. ** *p* < 0.01 (Student’s *t*-test).

To examine the effects of MG132 during NSC differentiation, we treated NSCs with MG132 for 48 h in the absence of growth factors. As shown in Figure 2A,B, MG132 (100 nM) increased the percentage of neurons while not affecting astrocytogenesis, as determined by ICC using TuJ1 and GFAP antibodies. When we counted the total cell numbers by nuclei, TuJ1 positive differentiated neurons, and GFAP positive astrocytes, MG132 significantly reduces the total cell number in a concentration-dependent manner and enhances neurogenesis at a concentration of 100 nM (Figure 2C,D). However, we did not observe an increase in astrocytogenesis by MG132 (Figure 2E). Moreover, 100 nM MG132 appeared to be toxic to astrocytes, as those were scanty after treatment at this concentration compared with astrocytes observed after treatment at other tested concentrations (Figure 2E). Western blotting data also revealed similar results that TUBB3 but not GFAP expression was induced by MG132 (100 nM) treatment (Figure 2F,G). These data suggested that MG132 (100 nM) increases the percentage of neurons in NSCs both in the presence and absence of mitogens while not affecting astrocytogenesis.

### 3.2. MG132 Reduces NSC Proliferation and Induces Cell Death

**Figure 3:**
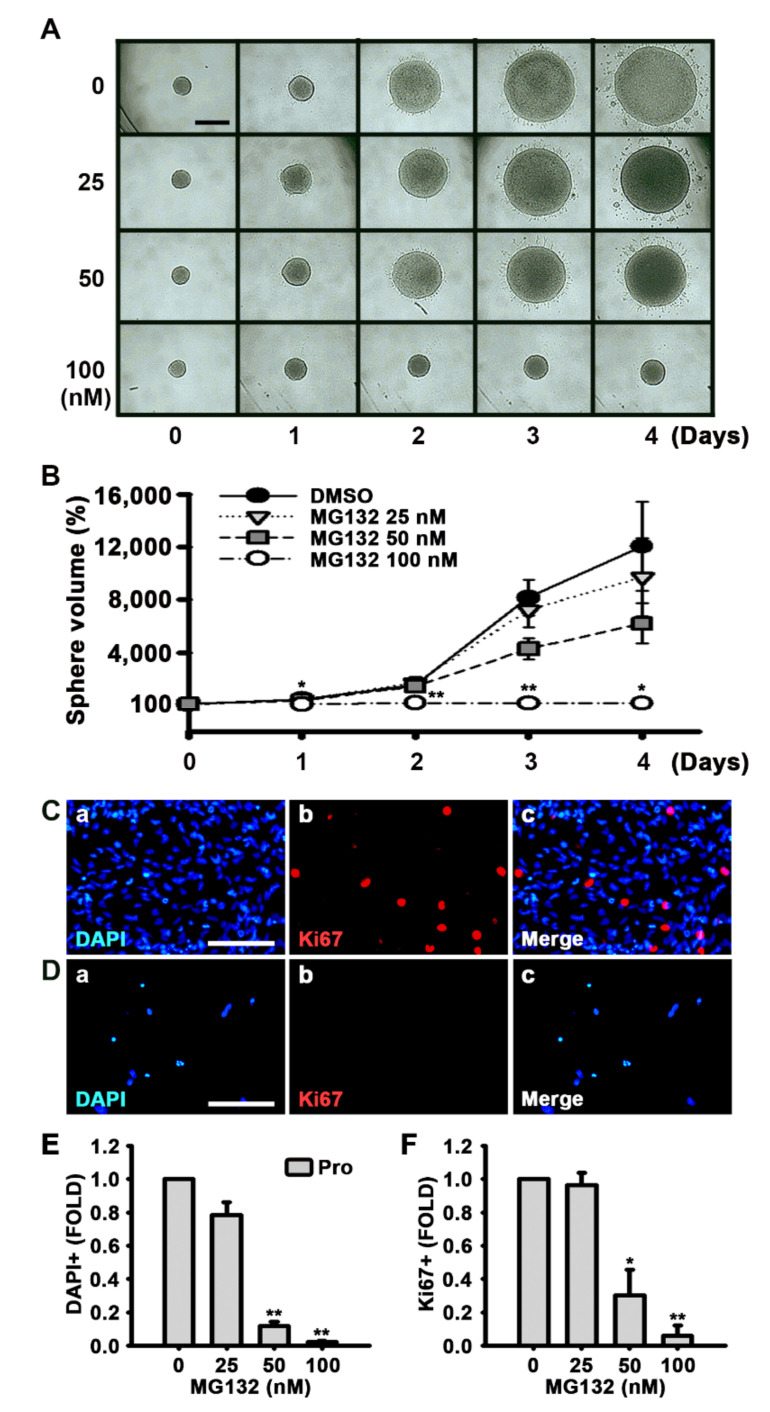
MG132 inhibits NSC proliferation in the presence of growth factors. (**A**) Digital images of neurospheres treated with either DMSO or 25, 50, and 100 nM MG132 for 4 days in the presence of EGF and FGF2. Scale bar = 500 µm. (**B**) Neurosphere volume was calculated by measuring the diameter of individual neurospheres treated with DMSO or MG132. Data are expressed as mean ± SEM (*n* = 3). (**C**,**D**) Representative immunofluorescence images of NSCs proliferated for 48 h. DAPI-positive nuclei (**a**, blue), Ki67-positive cells (**b**, red), and merged image (**c**). NSCs proliferated for 48 h with (**C**) DMSO or (**D**) 100 nM MG132 in the presence of EGF and FGF2 were immunostained with anti-Ki67 antibody. Scale bar = 100 µm. (**E**,**F**) Quantification of (**E**) nuclei and (**F**) Ki67-positive cells. Data are presented as mean ± SEM (*n* = 3). * *p* < 0.05, and ** *p* < 0.01, (Student’s *t*-test).

To investigate whether MG132 reduces NSC proliferation or MG132 is toxic to NSCs, we measured the size of neurospheres in the medium that promotes proliferation and contains EGF and FGF2 (Figure 3A,B). Phase contrast photographs were taken, and the diameters of the spheres were measured every other day, and the volumes of the neurospheres were calculated. As shown in Figure 3A,B, the size or volume of the neurospheres treated with 100 nM MG132 significantly decreased compared with that of the control.

**Figure 4:**
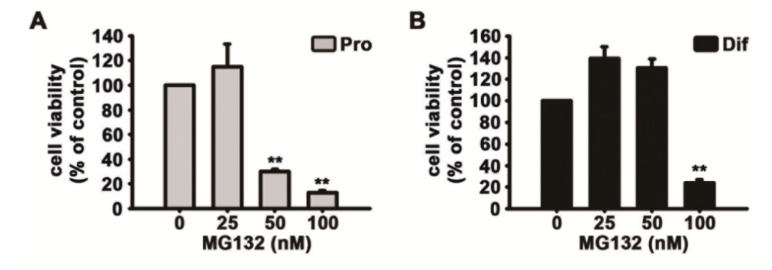
MG132 decreases cell survival during NSC proliferation and differentiation. (**A**,**B**) Cell viability was assessed using the 3-(4,5-dimethylthiazol-2-yl)-2,5-diphenyltetrazolium bromide assay. NSCs were treated with DMSO or various concentrations of MG132 (25–100 nM) for 2 days (**A**) in the presence or (**B**) absence of EGF and FGF2. Data are presented as mean ± SEM (*n* = 3). ** *p* < 0.01 (Student’s *t*-test).

Ki67 is found during all active stages of the cell cycle and is used as a proliferation marker [34]. MG132 significantly reduced both the number of cells detected by DAPI and Ki67-positive cells in a concentration-dependent manner following feeding of NSCs with the medium supplemented with growth factors (Figure 3C–F). In the MTT assay, we also observed that MG132 reduces the number of not only proliferated cells but also differentiated cells (Figure 4A,B). These results suggested that MG132 inhibits NSC proliferation even in the presence of mitogens and/or may be toxic to NSCs.

**Figure 5:**
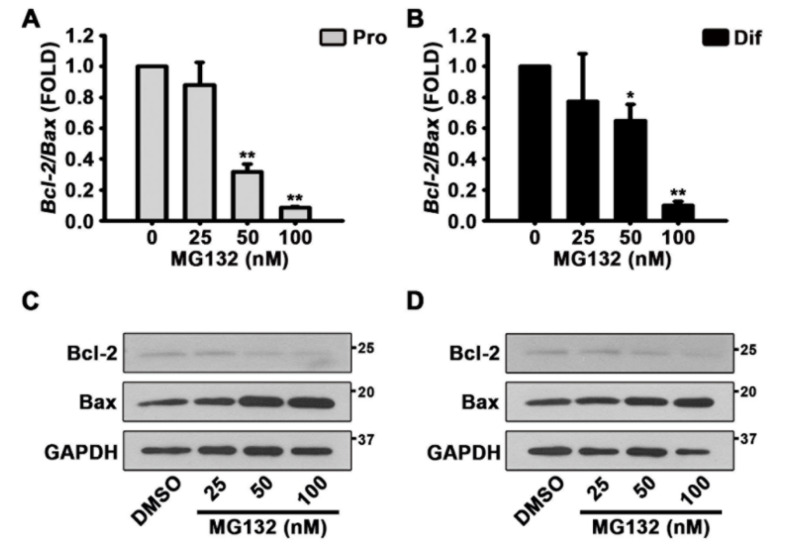
MG132 induces apoptosis by decreasing the B-cell lymphoma 2 (Bcl-2)/Bcl-2 associated X (Bax) ratio in NSCs. (**A**,**B**) After treatment with DMSO or various concentrations of MG132 (25–100 nM) for 48 h, total RNA from (**A**) proliferated or (**B**) differentiated NSCs was isolated and subjected to reverse transcription-polymerase chain reaction (RT-PCR) to quantify *Bcl-2* and *Bax* mRNA levels. *Gapdh* was used as an internal control. (**C**,**D**) Representative images of the protein bands of Bcl-2 and Bax. Two days after treatment, total cell lysates from (**C**) proliferated or, (**D**) differentiated NSCs were subjected to western blotting analysis with Bcl-2 and Bax antibodies. GAPDH was used as a loading control. Data are expressed as mean ± SEM (*n* = 3). * *p* < 0.05, and ** *p* < 0.01, (Student’s *t*-test).

Therefore, to assess whether MG132 triggers apoptosis, we performed qPCR and western blotting experiments to detect the levels of Bcl-2 and Bax transcripts and proteins, respectively (Figure 5A,D). The treatment of NSCs with MG132 both in the proliferation and differentiation conditions significantly reduced the transcript ratio of *Bcl-2/Bax* (Figure 5A,B). The protein levels of Bax were increased by MG132 in a concentration-dependent manner, whereas those of Bcl-2 decrease (Figure 5C,D and Figure S2). These data suggested that MG132 inhibits NSC proliferation and induces apoptosis.

### 3.3. MG132 Activates CREB Phosphorylation and Induces BDNF Expression

**Figure 6:**
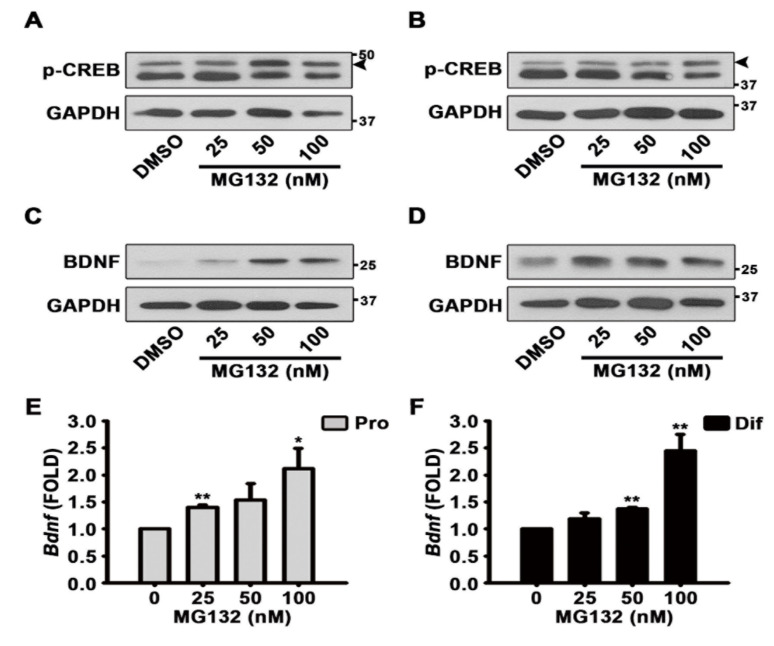
MG132 activates brain-derived neurotrophic factor (BDNF) through phosphorylation of cAMP response element-binding protein (CREB). (**A**–**D**) Representative images of the protein bands of (**A**,**B**) phospho-CREB and (**C**,**D**) BDNF. Two days after treatment, total cell lysates from (**A**,**C**) proliferated or (**B**,**D**) differentiated NSCs were subjected to western blot analysis with phospho-CREB and BDNF antibodies. GAPDH was used as a loading control. (**E**,**F**) After treatment with DMSO or various concentrations of MG132 (25–100 nM) for 48 h, total RNA from (**E**) proliferated or (**F**) differentiated NSCs was isolated and subjected to RT-PCR for quantification of *Bdnf* mRNA levels. *Gapdh* was used as an internal control. Data are presented as mean ± SEM (*n* = 3). * *p* < 0.05, and ** *p* < 0.01 (Student’s *t*-test).

To identify the mechanisms by which MG132 (100 nM) increases the percentage of neurons although MG132 is toxic, we performed western blotting experiments to determine whether CREB is activated in NSCs upon MG132 treatment. When NSCs were cultured in the presence (Figure 6A and Figure S3) and absence (Figure 6B and Figure S3) of EGF and FGF2, MG132 activated CREB phosphorylation. We detected the expression of BDNF following MG132 treatment, and the results showed that in the presence of growth factors, MG132 induces BDNF expression (Figure 6C and Figure S4). Similarly, in the absence of mitogens, MG132 increased BDNF expression (Figure 6D and Figure S4). Because MG132 is a proteasome inhibitor, it is possible that the induction of BDNF expression could be simply due to the inhibition of protein degradation. Therefore, to investigate whether MG132 induces *Bdnf* gene expression, qPCR was performed after RT-PCR. As shown in Figure 6E,F, MG132 induced *Bdnf* transcripts in NSCs cultured in both proliferation and differentiation conditions. These data suggested that MG132 activates CREB phosphorylation and induces BDNF transcript and protein expression.

## 4. Discussion

Controlling protein synthesis and regulating protein degradation are important processes in the maintenance of cell survival and function. Recent evidence suggests that stem cell function and biology can be modulated by proteasomes [43,44,45]. Although still controversial, it has been reported that the activities and expression of proteasomes decline as cultured cells or animals grow old [46]. Given that protein synthesis and removal have to be finely tuned for normal cell function, impaired protein degradation due to reduced proteasome function would have detrimental effects on the cell. The increase and accumulation of proteins are a feature of neurodegenerative diseases, and cancer cells utilize proteasomes for their survival by removing tumor suppressor proteins, such as p53 [11]. We recently discovered that astrocytogenesis is regulated by histone demethylase KDM5A, which is regulated at the translational level [47]. Therefore, the control of protein expression appeared to be important for NSC differentiation and biology. Our current study showed that the proteasome inhibitor MG132 inhibited NSC proliferation and elevated neuron percentage. Treatment with MG132 increased CREB phosphorylation and BDNF levels. Interestingly, the effects of MG132 on BDNF upregulation appeared to be mediated by the induction of gene transcription, as not only the protein levels but also the mRNA levels of BDNF were increased.

Recent data suggest that not only neurons but also astrocytes are essential players for neurodegenerative diseases [48,49]. Astrocytes are essential for proper neuronal functions such as synapse formation and maturation, amino acid catabolism [50,51]. In neurodegenerative diseases, astrocytes are activated to proliferate and upregulate gene expression due to inflammation and cell damage [52]. Although these reactive astrocytes result in scar formation and inhibit axonal growth, it has been reported that these cells also provide protective effects by removing excitotoxic glutamate and beta amyloids [52]. During neurodegenerative disease progression, alteration of proteasome composition has been observed in glial cells [53,54]. These suggest that astrocyte activation during neurodegenerative diseases may be beneficial for removing pathological waste proteins and astrocytes may be important therapeutic targets [49].

Astrocytes can be generated by cytokines including CNTF by activation of signal transducer and activator of transcription 3 and Sma- and Mad-related proteins [3]. Interestingly, cytokines that are known to induce inflammation enhances astrocytogenesis [3]. In addition, brain damages associated with hypoxia result in astrocyte activation [55]. Therapeutic hypothermic intervention after hypoxia in human primary cortical astrocytes showed specific transcriptomes and proteome profiles [56]. It is also noted that gap junctions and hemichannels connects neurons and glia, and shares signals, and provide buffering of toxicity [57]. Controversially, damaging signals can be transferred to adjacent neurons, thereby increasing neuronal death and inflammation [57]. To mediate various functions of neurons, high energy is needed [58]. Glia cooperates with neurons to orchestrate the metabolic reactions for proper neuronal activity [58]. Therefore, further studies are needed to unravel the roles of glia in neurodegenerative diseases.

It has been known that treatment with MG132 inhibits proteasomes and can mimic the aged status of cells [45]. Our study showed that MG132 treatment was toxic to NSCs and decreased the Bcl-2/Bax ratio. Similarly, it has been reported that in aged mice with low proteasome activity, the proliferation and self-renewal of neural progenitor cells (NPCs) are reduced [45]. However, in contrast to our finding that MG132 increases neuron percentage, another study showed that the number of neurons was reduced in NPCs derived from aged mice at postnatal day 90 (P90) and P540 [45]. It is possible that the increase in neuron percentage by MG132 in our study was due to the decreased total cell numbers, which was caused by the toxicity of MG132. However, impairment of proteasome function by MG132 increased *Bdnf* transcripts. Because MG132 inhibits protein degradation, we expected it to upregulate BDNF protein level; thus, the observed activation of *Bdnf* gene transcription was somewhat surprising. As MG132 was toxic to NSCs, the cells might have increased BDNF expression as a compensatory protective mechanism. As a result, neurons were the least affected cells when NSCs were treated with MG132 and induced to differentiate. Similar to our results, it has been reported that pluripotent stem cells are extraordinarily sensitive to MG132, whereas motor neurons were relatively resilient to MG132 [59]. Furthermore, it is possible that inhibition of proteasome activity and function might be more toxic to undifferentiated NSCs and other differentiated cells than it might affect differentiated neurons [59]. MG132 is a potent tripeptide aldehyde (benzyloxycarbonyl Leu-Leu-Leu-aldehyde; ZLLLal) proteasome inhibitor [11]. In the early 1990s, treatment with ZLLLal was reported to induce long neurite outgrowth in PC12 cells and was suggested to induce neurogenesis [60]. Recently, in a stable cell line that expresses mutated TAR DNA-binding protein-43 to mimic the pathology of amyotrophic lateral sclerosis, MG132 treatment promoted neurite extension [61]. Therefore, in our study, it is also possible that MG132 treatment may have increased neurogenesis in NSCs during differentiation. pCREB is observed in newly generated hippocampal granule neurons and concurrently detected with doublecortin, but it diminishes as neurons mature [62,63]. Using a dominant-negative CREB mutant, it has been identified that CREB signaling is essential for the survival and differentiation of newborn neurons [62,63,64]. In addition, CREB phosphorylation is known to increase BDNF expression [65,66,67]. In our study, we detected the activation of CREB phosphorylation and the induction of BDNF expression upon MG132 treatment. Therefore, CREB phosphorylation by MG132 treatment appeared to be responsible for the increased BDNF expression and survival of neurons. Although MG132 was toxic to NSCs and inhibited proliferation, we observed that MG132 increased the percentage of neurons during NSC differentiation. It is interesting to study that other proteasome inhibitors, although they might be toxic, can also induce neurogenesis or increase neuron survival in NSCs. Therefore, it would be interesting to modify the chemical structure of MG132 to develop proteasome inhibitors with reduced toxicity but with the ability to induce neurogenesis or protect neurons in the treatment of neurodegenerative diseases.

## 5. Conclusions

**Figure 7:**
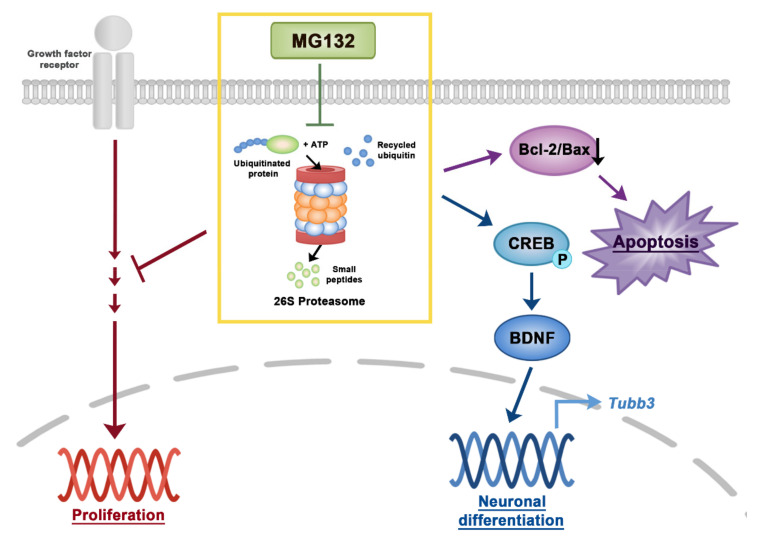
A scheme of signaling pathways in NSCs induced by MG132. Inhibition of proteasome by MG132 induced apoptosis by decreasing the Bcl-2/Bax ratio in NSCs. In addition, MG132 increases neuronal differentiation or survival in NSCs by activating CREB and BDNF. Proteasome inhibition by MG132 inhibited NSC proliferation in the presence of growth factors.

The proteasome inhibitor MG132 reduced NSC proliferation and was toxic to NSCs (Figure 7). However, MG132 treatment increased the percentage of neurons and induced phosphorylation of CREB and expression of BDNF transcripts and proteins (Figure 7). Our data revealed the importance of proteasome in the regulation of NSC proliferation and differentiation. By modifying MG132 chemical structure, less toxic and more efficient neuroprotective or neurogenic chemicals can be developed to treat neurodegenerative diseases.

## Supplementary Materials

The following are available online at https://www.mdpi.com/2218-273X/10/11/1507/s1, Figure S1: Uncropped 3 batches image of western blots for the TUBB3 and GFAP, Figure S2: Uncropped 3 batches image of western blots for the Bcl-2 and Bax, Figure S3: Uncropped 3 batches image of western blots for the p-CREB, Figure S4: Uncropped 3 batches image of western blots for the BDNF.
