# Supplementary material for: Proteasome Inhibitor MG132 is Toxic and Inhibits the Proliferation of Rat Neural Stem Cells but Increases BDNF Expression to Protect Neurons
Source: Biomolecules. 2020 Nov 2;10(11):1507. doi: 10.3390/biom10111507 (PMC7692322; doi:10.3390/biom10111507)
Supplement: Supplementary file 1 [file biomolecules-10-01507-s001.pdf]

Supplementary figures

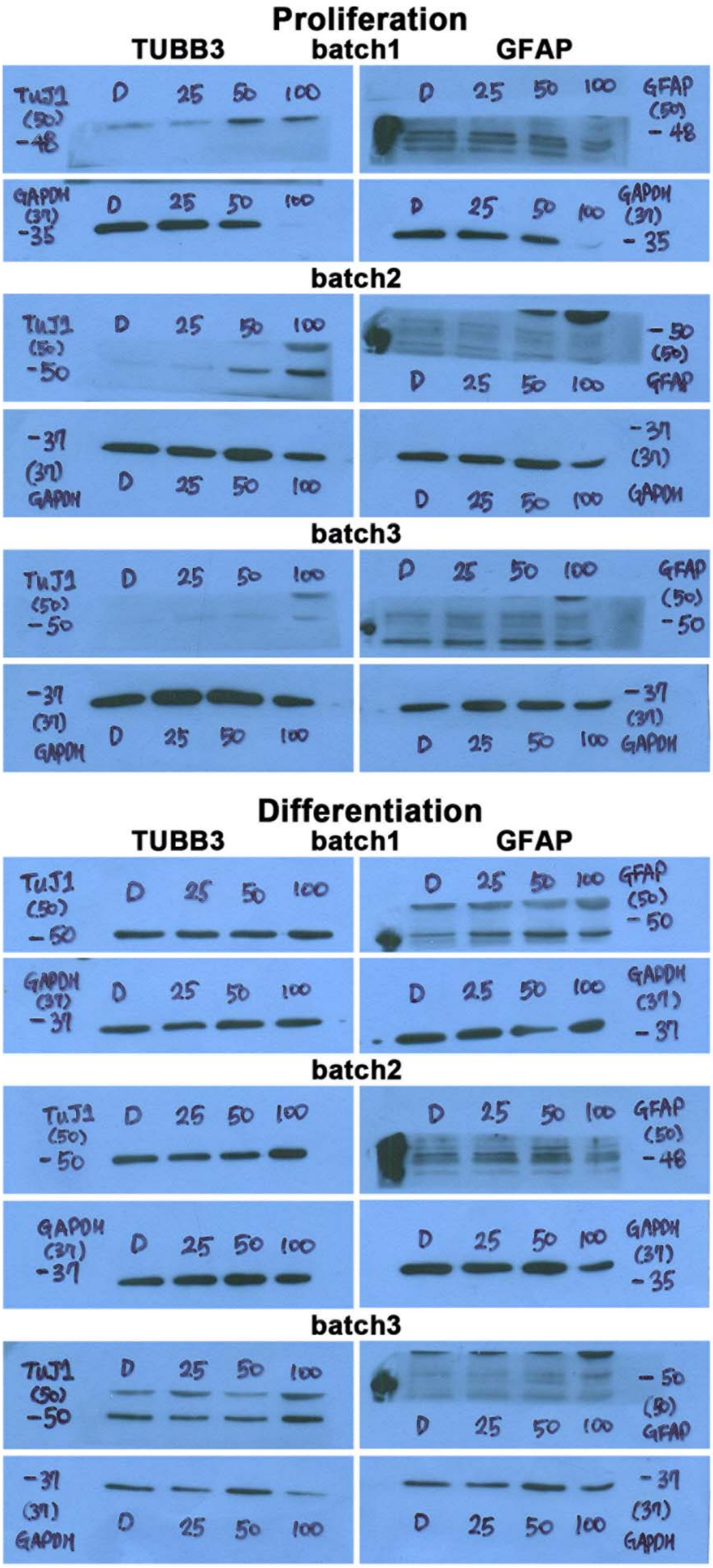

Figure S1. Uncropped 3 batches image of western blots for the TUBB3 and GFAP.

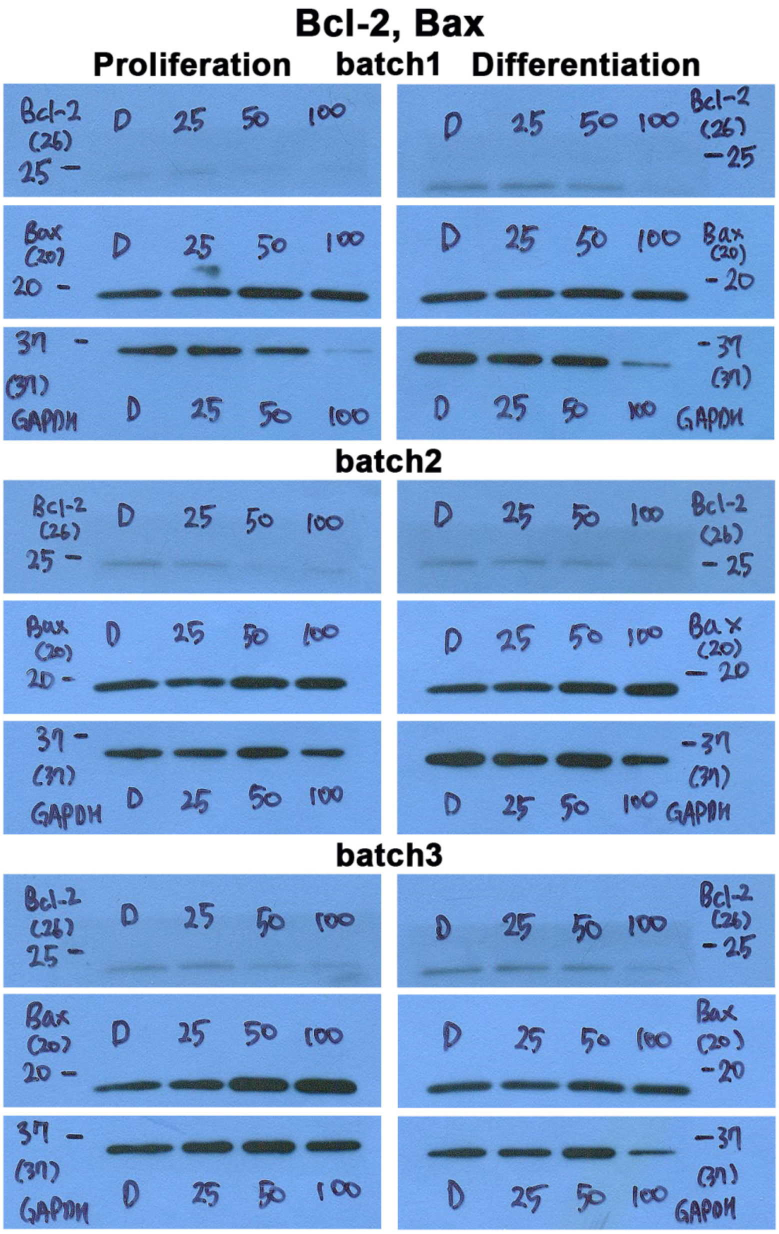

Figure S2. Uncropped 3 batches image of western blots for the Bcl-2 and Bax.

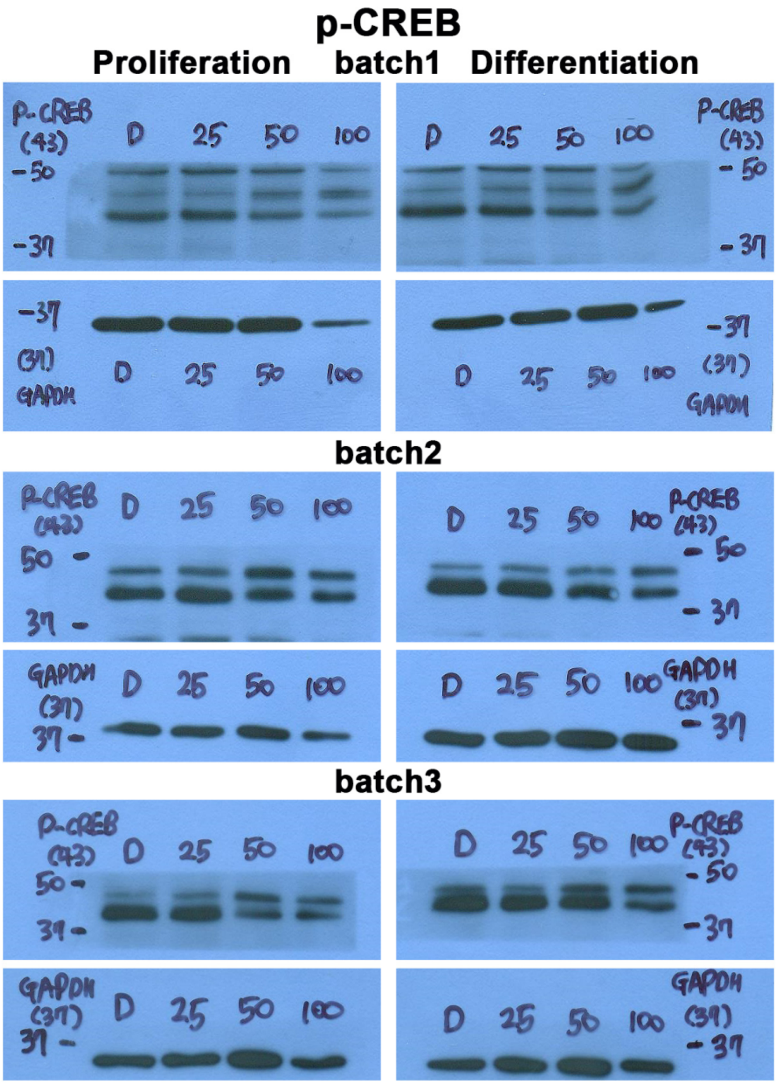

Figure S3. Uncropped 3 batches image of western blots for the p-CREB.

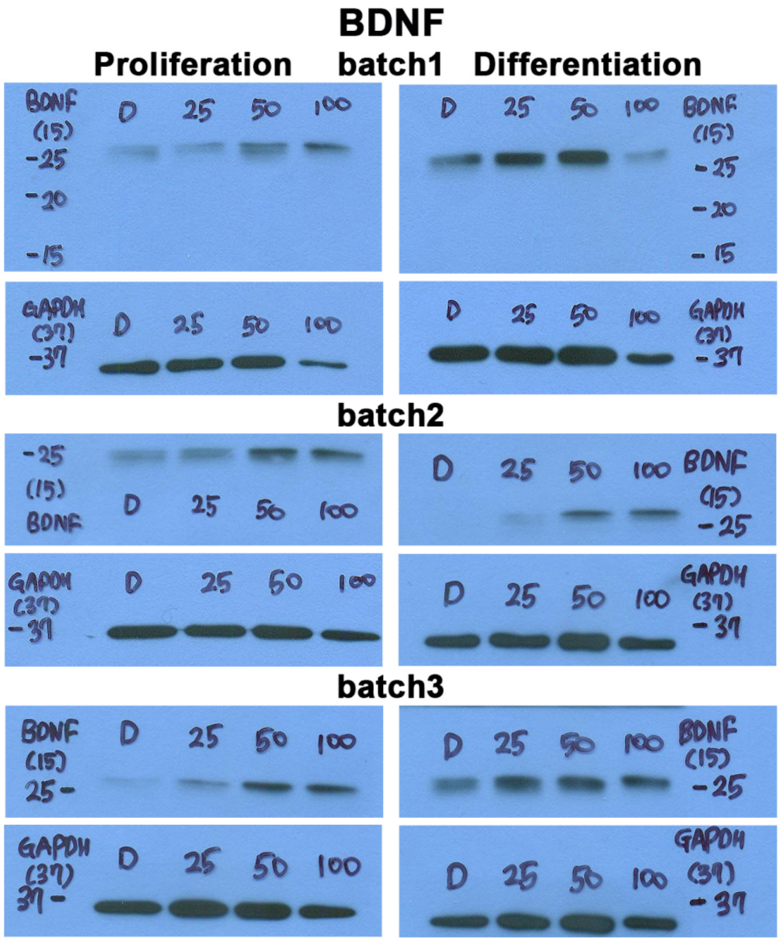

Figure S4. Uncropped 3 batches image of western blots for the BDNF.
